# Clinical observation of stapokibart in patients under 18 years of age with seasonal allergic rhinitis: a case series study of 20 cases

**DOI:** 10.3389/falgy.2026.1785270

**Published:** 2026-02-27

**Authors:** Long-fei Wang, Wei-cong Ma, Chen-xing Kan, Guo-dong Hao

**Affiliations:** 1 Department of Respiratory Medicine, Graduate School of Tianjin Medical University, Tianjin, China; 2 Department of Allergy, Tangshan Workers Hospital, Tangshan, Hebei, China

**Keywords:** atopic dermatitis, children, interleukin-13, interleukin-4, seasonal allergic rhinitis, stapokibart

## Abstract

**Objective:**

The objective of this work was to investigate the efficacy and safety of stapokibart, an anti-IL-4R*α* monoclonal antibody, in patients under 18 years of age with moderate-to-severe seasonal allergic rhinitis (SAR).

**Methods:**

A retrospective analysis was conducted on 20 children with severe SAR who received stapokibart treatment during spring (March–April) and autumn (August–October) of 2025. The regimen consisted of an initial dose of 600 mg (two subcutaneous injections), followed by a maintenance dose of 300 mg (one injection) after 2 weeks, after which the treatment was discontinued. Children with comorbid atopic dermatitis (AD) (*n* = 8) were treated according to the AD protocol. The primary efficacy endpoint was the change in visual analog scale (VAS) scores for nasal symptoms after 1 month of treatment compared with baseline. Secondary endpoints included symptom control during the allergic season and drug safety.

**Results:**

All 20 pediatric patients demonstrated significant reductions in nasal VAS scores compared with baseline after 1 month of treatment (*P* < 0.001). Among the eight patients with comorbid AD, no clinically significant exacerbations of SAR symptoms occurred during subsequent allergy seasons during continued treatment. Only one patient (5%) reported mild drowsiness as an adverse event, with no severe adverse events observed.

**Conclusion:**

In this small case series, stapokibart rapidly and effectively alleviated nasal symptoms of SAR in children and adolescents, and may provide long-term symptom control across allergy seasons for patients with comorbid AD. The treatment demonstrated a good safety profile, offering novel insights into targeted therapy of comorbid allergic diseases in children.

## 1 Introduction

Seasonal allergic rhinitis (SAR) is one of the most common allergic diseases in children and adolescents, characterized primarily by nasal itching, sneezing, watery nasal discharge, and nasal congestion (1). It severely affects learning efficiency, sleep quality, and overall quality of life (2). Traditional treatments include nasal corticosteroids, oral antihistamines, and leukotriene receptor antagonists. However, some patients with moderate-to-severe conditions may experience suboptimal symptom control or difficulty tolerating adverse drug reactions.

Stapokibart is a fully human monoclonal antibody that blocks the signaling pathways of interleukin-4 (IL-4) and interleukin-13 (IL-13) through high-affinity binding to the IL-4R*α* subunit (3). These two cytokines are core drivers of type 2 inflammation and play a pivotal role in the pathogenesis of both severe atopic dermatitis (SAR) and atopic dermatitis (AD) (4). Currently, stapokibart has been approved in China for the treatment of moderate-to-severe atopic dermatitis (AD) in patients aged 12 years and above, as well as for seasonal allergic rhinitis in adults. However, clinical experience with stapokibart in seasonal allergic rhinitis among patients under 18 years of age—particularly in pediatric cases and those with allergic comorbidities—remains limited.

The present study aimed to preliminarily evaluate the short-term efficacy, cross-seasonal control potential, and safety of stapokibart in 20 pediatric patients under 18 years of age with seasonal allergic rhinitis. By analyzing clinical data from this cohort, the study aimed to provide empirical references to guide clinical practice.

## 2 Materials and methods

### 2.1 Study subjects

A total of 20 pediatric patients diagnosed with SAR were enrolled from the Department of Allergy between January and October 2025. Inclusion criteria: (1) age 6–18 years; (2) diagnosis of SAR confirmed by allergen testing, with primary sensitization to spring pollen (e.g., cypress, birch) and/or autumn pollen (e.g., mugwort, foxtail) allergens; (3) nasal symptom VAS score ≥7 (moderate to severe); and (4) provision of informed consent and treatment with stapokibart. Exclusion criteria: (1) allergy to biologic components, (2) presence of active infection, and (3) severe dysfunction of vital organs.

### 2.2 Therapeutic regimen

#### 2.2.1 Basic regimen (12 cases of isolated SAR)

Patients received the first dose of stapokibart 600 mg (two vials, subcutaneous injection), followed by a second dose of 300 mg (one vial) after 2 weeks. Treatment was discontinued after completion of these two doses. Therapy was initiated either before the allergy season or at the initial onset of symptoms.

#### 2.2.2 Comorbidity regimen (eight cases of severe to moderate AD in SAR)

Patients received the same first two were administered as the basic regimen, followed by continuous treatment with the standard maintenance regimen of stapokibart for AD (300 mg every 4 weeks), with the duration of treatment determined by the severity of AD.

### 2.3 Observation indicators

#### 2.3.1 Primary efficacy endpoint

The primary endpoint was the change in the total VAS score for nasal symptoms (including nasal congestion, rhinorrhea, nasal pruritus, and sneezing) from baseline to 1 month after initiation of treatment.

#### 2.3.2 Secondary efficacy endpoint

During follow-up in subsequent allergy seasons (spring or autumn), recurrence of SAR symptoms was recorded (defined as a VAS score increase of ≥3 points compared with the remission phase).

#### 2.3.3 Safety indicators

All adverse events occurring during treatment were recorded. Particular attention was given to injection site reactions, conjunctivitis, drowsiness, and headache.

### 2.4 Statistical methods

Data were analyzed using SPSS version 26.0 software. Changes in VAS scores before and after treatment were compared using paired t test. A *P*-value <0.05 was considered statistically significant.

## 3 Bear fruit

### 3.1 General data

**Figure 1:**
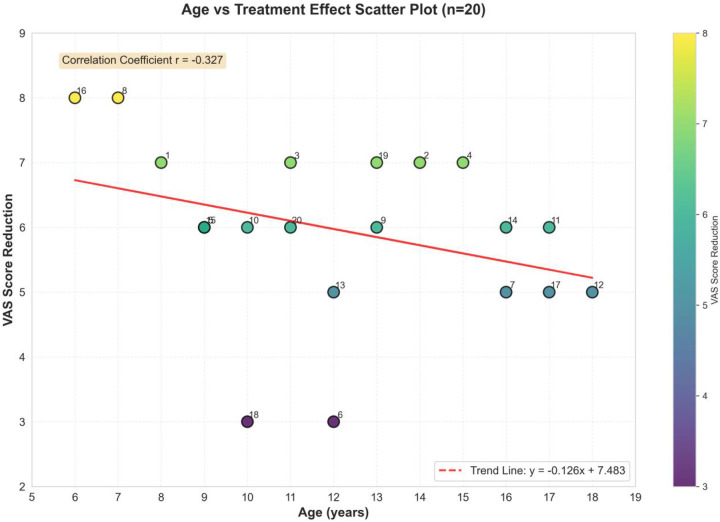
A scatter plot, representing a trend plot of treatment outcomes.

**Figure 2:**
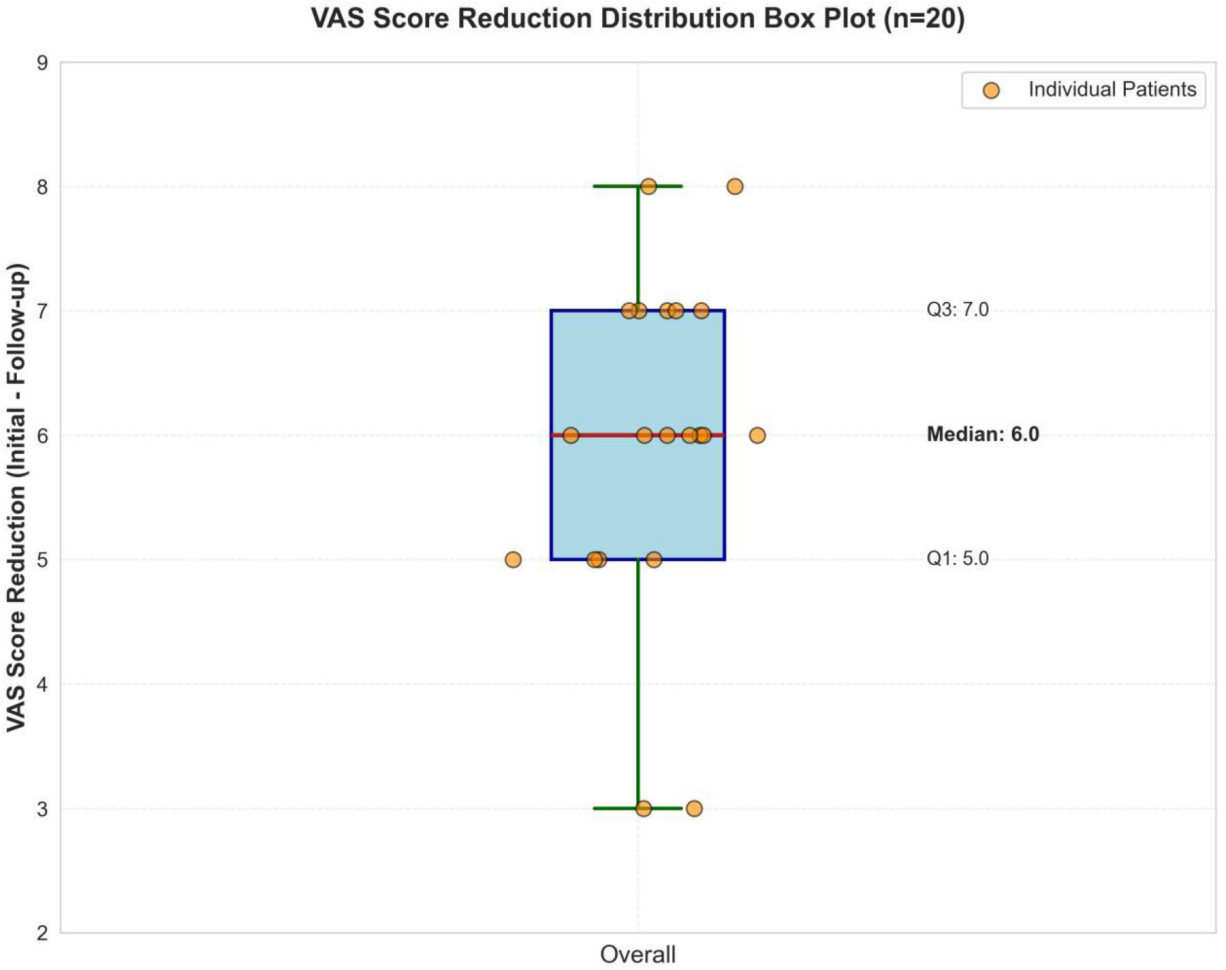
A box plot representing treatment improvement symptoms.

Among the 20 pediatric patients, 12 were male and 8 were female, with an average age of 12.1 ± 3.5 years. Among them, eight patients (40%) had comorbid moderate-to-severe atopic dermatitis (Figures. 1, 2).

### 3.2 Efficacy analysis

#### 3.2.1 Short-term efficacy

**Figure 3:**
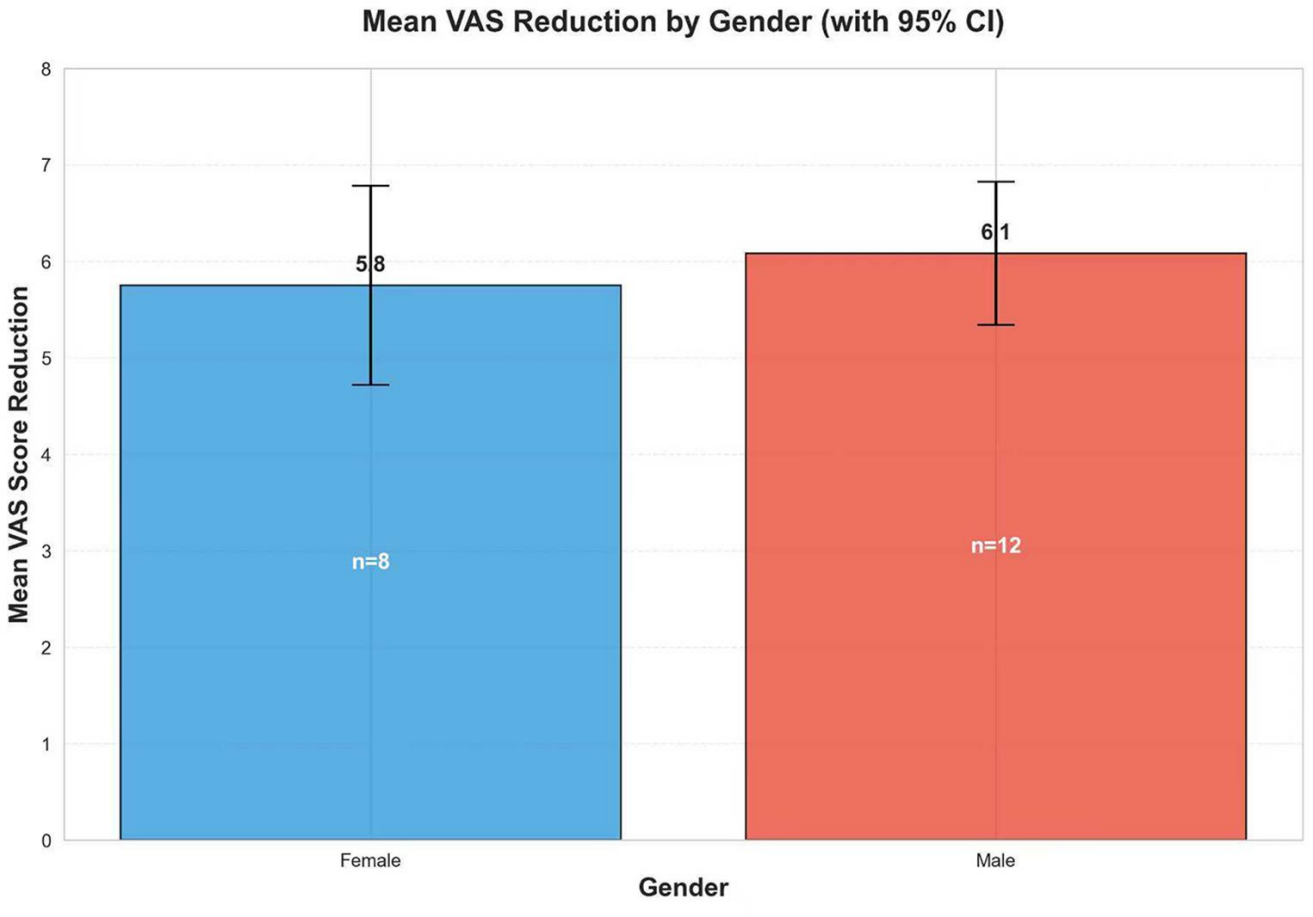
A gender symptom comparison.

**Figure 4:**
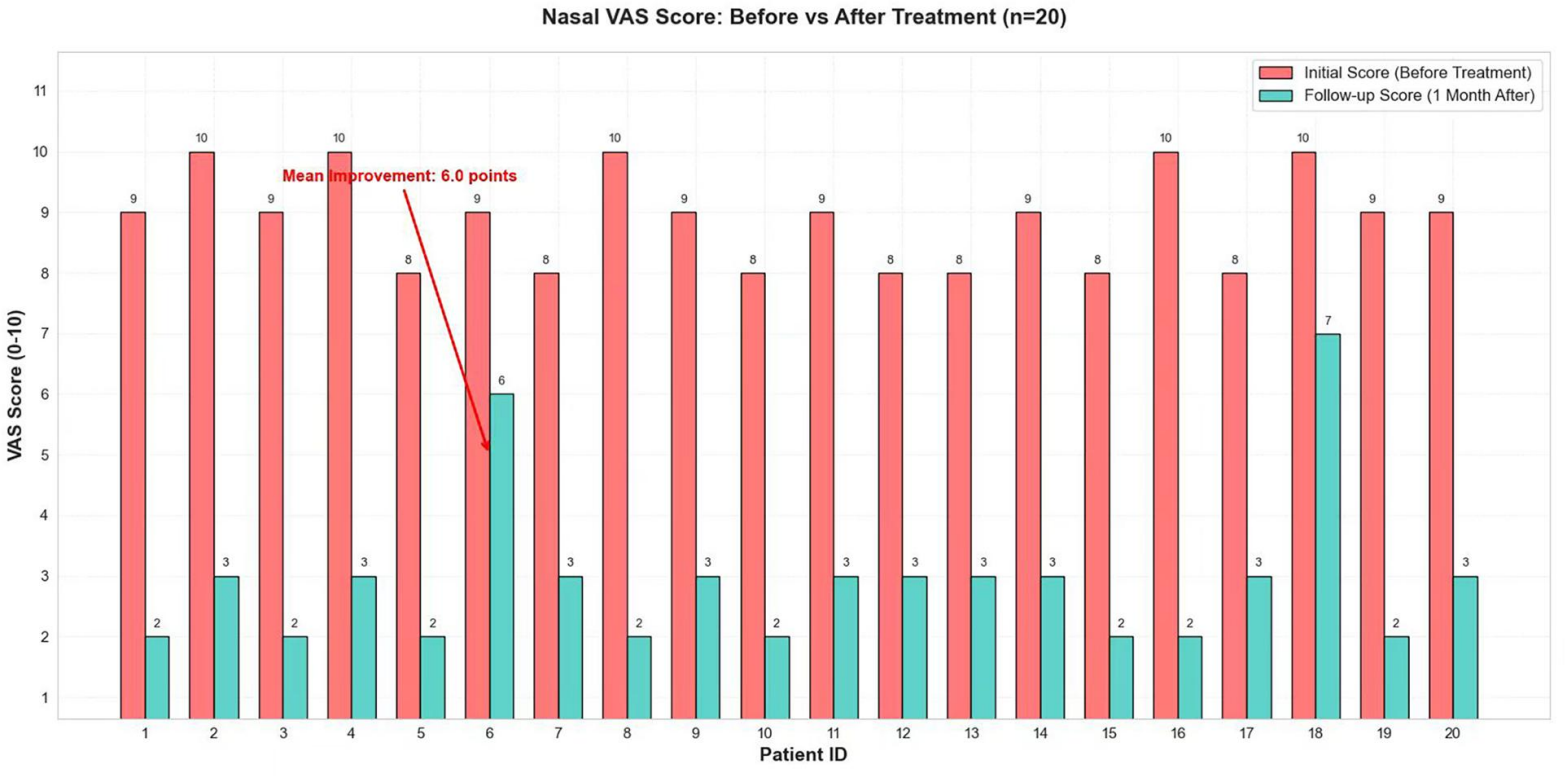
The comparison of VAS scores before and after treatment.

Age had minimal influence on treatment effect. Within the 6–18-year age range, no significant correlation was observed between age and efficacy (r = −0.32, *P* = 0.17; Figure 3). After 1 month of treatment, the nasal VAS scores of all 20 pediatric patients decreased significantly from baseline (8.9 ± 0.7) to (2.9 ± 1.2) (t = 19.85, *P* < 0.001; Table 1). The mean VAS score reduction following treatment was 6.0 points, with an improvement rate of 67.4% (Figure 4), demonstrating a highly statistically significant difference.

**Table 1:** Results of statistical tests.

Inspecting item	Method of calibration	Statistics	*P* price	Statistical significance	Clinical significance
Pre- and post-treatment comparison	Paired t test	t = 19.85	<0.001	Significantly	Effective treatment
Differences in scores before and after treatment	Effect size (Cohen’s d)	d = 4.44	—	Maximal effect	Significant clinical implications

#### 3.2.2 Long-term seasonal control

Cross-seasonal follow-up (mean follow-up duration of 6 months) was conducted for eight pediatric patients with comorbidities who received continuous treatment according to the AD protocol. During subsequent allergy seasons, none of these patients experienced clinically significant exacerbations of SAR, and nasal symptoms remained at mild levels (VAS <3).

### 3.3 Safety analysis

All pediatric patients tolerated the treatment well. Only one patient (5%) reported mild drowsiness after the first injection, which resolved spontaneously after discontinuation and did not affect subsequent treatment. No injection site reactions, conjunctivitis, headache, or other systemic adverse events were observed. There were also no cases of treatment discontinuation due to adverse reactions.

## 4 Discussion

This study is the first to observe rapid and significant improvement in nasal symptoms following short-term (two-dose) stapokibart treatment in a small cohort of pediatric patients under 18 years of age with SAR. The observed effect is attributed to the drug’s potent inhibition of the IL-4/IL-13 pathway, thereby blocking the allergen-induced type 2 inflammatory cascade. Notably, the onset of action may be faster than that of conventional drugs, providing a novel option for children requiring rapid symptom control.

Notably, among the eight pediatric patients with comorbid AD, sustained treatment with stapokibart prevented clinical exacerbations during subsequent natural allergen exposure seasons. This strongly suggests that continuous targeted inhibition of the shared inflammatory pathway in type 2 immunity not only controls AD skin lesions but also exerts long-term, cross-seasonal upstream control over respiratory allergies. These results demonstrate the therapeutic advantage of the one-pathway, multiple-diseases concept, potentially shifting treatment strategies for children with allergic comorbidities from organ-specific symptomatic management to systemic disease management.

In terms of safety, the incidence of adverse reactions in this group of pediatric patients was extremely low, with only one patient exhibiting transient drowsiness, which is significantly lower than the common central nervous system adverse reactions such as drowsiness and fatigue associated with conventional oral antihistamines. This finding highlights the high selectivity advantage of targeted biologic therapy, which is particularly crucial for pediatric and adolescent patients who require focused attention for learning.

### 4.1 Limitations of this study

As a retrospective, single-center, small-sample case series, this study lacks a control group and has a relatively short follow-up period. The time to symptom rebound after discontinuation of medication, optimal dosing cycles, and long-term safety in children with isolated SAR still require further clarification through large-scale, prospective randomized controlled trials.

## 5 Conclusion

Stapokibart demonstrated rapid and significant improvement in nasal symptoms among children under 18 years of age with SAR, with a favorable safety profile. For children with comorbid AD, continued treatment may achieve long-term, cross-seasonal control of SAR. This study provides preliminary clinical evidence for the application of stapokibart in the field of pediatric allergic comorbidities, supporting its use as one of the preferred targeted treatment options for children with moderate-to-severe comorbid conditions.

## Data Availability

The raw data supporting the conclusions of this article will be made available by the authors, without undue reservation.
